# Instrumented Mouthguards in Women’s Rugby League: Quantifying the Incidence and Probability of Head Acceleration Events at Group and Individual Levels

**DOI:** 10.1007/s40279-026-02420-9

**Published:** 2026-04-10

**Authors:** James Tooby, Sean Scantlebury, Cameron Owen, Mily Spiegelhalter, Kathryn Dane, Carolyn A. Emery, Matthew Kitchin, Gemma Phillips, Thomas Sawczuk, Isla Shill, Kevin Till, Dane Vishnubala, Ben Jones

**Affiliations:** 1 Carnegie Applied Rugby Research (CARR) Centre, Carnegie School of Sport, Leeds Beckett University, Leeds, UK; 2 Rugby Football League, Etihad Campus, Manchester, UK; 3 Leeds Rhinos Rugby League Club, Leeds, UK; 4 Sports Injury Prevention Research Centre, Faculty of Kinesiology, University of Calgary, Calgary, AB Canada; 5 Human Performance Laboratory, Faculty of Kinesiology, University of Calgary, Calgary, AB Canada; 6 Alberta Children’s Hospital Research Institute, University of Calgary, Calgary, AB Canada; 7 McCaig Institute for Bone and Joint Health, University of Calgary, Calgary, AB Canada; 8 O’Brien Institute for Public Health, University of Calgary, Calgary, AB Canada; 9 Hotchkiss Brain Institute, University of Calgary, Calgary, AB Canada; 10 Department of Community Health Sciences, Cumming School of Medicine, University of Calgary, Calgary, AB Canada; 11 Department of Pediatrics, Cumming School of Medicine, University of Calgary, Calgary, AB Canada; 12 Hull Kingston Rovers Rugby League Club, Hull, UK; 13 Obesity Institute, Leeds Beckett University, Leeds, UK; 14 Edinburgh Sports Medicine Research Centre, Institute for Sport, PE and Health Sciences, University of Edinburgh, Edinburgh, UK; 15 UK Collaborating Centre On Injury and Illness Prevention in Sport, University of Edinburgh, Edinburgh, UK; 16 University of Leeds, Leeds, UK; 17 Division of Physiological Sciences and Health Through Physical Activity, Lifestyle and Sport Research Centre, Department of Human Biology, Faculty of Health Sciences, University of Cape Town, Cape Town, South Africa; 18 School of Behavioural and Health Sciences, Faculty of Health Sciences, Australian Catholic University, Brisbane, QLD Australia; 19 PREM Rugby, London, UK

## Abstract

**Background:**

Head acceleration event (HAE) exposure is a concern in sport owing to potential effects on brain health. Despite growth in the sport's growing in popularity, HAE exposure in women’s rugby league has yet to be quantified.

**Objectives:**

The aim of the study was to examine HAE incidence and probability across Women’s Super League rugby league players, including position- and player-specific HAE incidence and probability.

**Methods:**

Instrumented mouthguards (iMGs) were worn by 136 players during the 2024 season, across 48 video-analysed matches, resulting in 568 player matches with iMG data. The incidence of HAEs and the probability of HAEs from ball-carries and tackle attempts were estimated using generalised linear mixed models and average positions on an individual-player basis.

**Results:**

The average incidence of HAEs exceeding 25 g ranged from 0.40 to 0.65 per median playing time for back positions and 0.54 to 0.66 for forward positions. The probability of recording an HAE exceeding 25 g during a ball-carry was 1.33% and a tackle-attempt was 1.28%. Some individuals had higher HAE incidence and probability compared with position group means (e.g. one player exhibited an average of 1.77 HAEs exceeding 25 g per match, over double the average for their position).

**Conclusions:**

This study quantifies HAE incidence and probability in women’s rugby league match-play, allowing for the comparison of HAE exposure with other sports. Overall, HAE incidence is lower than previously reported for men’s rugby league and for women’s rugby union. However, given elevated HAE incidence in some players, continued HAE monitoring using iMGs is necessary for managing the potential risks of HAE exposure.

**Supplementary Information:**

The online version contains supplementary material available at 10.1007/s40279-026-02420-9.

## Key Points

**Table:** 

The mean incidence of head acceleration events (HAEs) is provided using a range of magnitude thresholds for each position group and on an individual-player basis in Women’s Super League for the first time, revealing lower average HAE incidence than previously reported in men’s rugby league and women’s rugby union.
The probability of HAEs being recorded during ball-carries and tackle attempts was also lower than previously reported in men’s rugby league and women’s rugby union.
Some players exhibited elevated HAE incidence and probability values with respect to the position group averages, which raises concern over individuals' accumulated HAE exposure.

## Introduction

Women’s rugby league has grown globally in recent years [1], and elite competitions have been established in England (i.e. Women’s Super League; WSL), and Australasia (i.e. Women’s National Rugby League). Despite this growth, a call to action has highlighted the need to increase the evidence base (e.g. injury surveillance and injury risk factors) in women’s rugby league to inform best practices in injury prevention [2]. Since then, research studies, including women’s rugby league cohorts, have identified concussion as a major concern [3–6]. For example, the concussion rate in WSL has been reported at 8–14 concussions per 1000 player match-hours [3], similar to men’s rugby league (10–15 per 1000 player match-hours [3, 7]) and greater than rates in women’s rugby union (4–6 concussions per 1000 player match-hours) [8]. Head-to-head contacts have been identified as a major risk factor for concussion [4] and have been reported to occur in around 30% of tackle events [5], prompting interventions aimed at mitigating head-to-head contacts to reduce brain injury risk [9].

In addition to concussion, head acceleration events (HAEs) are also a growing concern in sports. The consensus definition of an HAE is an acceleration response of the head following a short-duration external collision force [10], and there is a potential dose–response relationship between repetitive HAE exposure and long-term health outcomes (e.g. neurological disorders [11, 12], chronic traumatic encephalopathy [13, 14]). Instrumented mouthguards (iMGs) have emerged as effective tools for providing objective estimates of the magnitude and frequency of HAEs during training and match-play [15], owing to their embedded inertial sensors and tight coupling with the skull [16]. Adoption of iMGs has been widespread in elite-level rugby, resulting in numerous studies investigating HAE exposure within men’s and women’s rugby union [17–20] and men’s rugby league [21, 22]. However, to date, no study has investigated HAE exposure in women’s rugby league.

Quantifying HAE exposure in women’s rugby league is crucial owing to the unique challenges and physiological differences faced by female players. A recent Delphi study identified several barriers specific to women’s rugby league, including poor tackle technique, lack of pre-season intensity, short training sessions, suboptimal medical standards, and limited access to physiotherapists [23]. These factors may significantly influence tackle technique and, consequently, may affect HAE exposure. Furthermore, research on sex differences in rugby union players has revealed that female athletes exhibit 47% lower isometric neck strength compared with males, and experience substantially different head-impact mechanisms and kinematics [24]. These findings strongly suggest that male-derived training and injury-prevention strategies may not be appropriate for female rugby athletes. Given these sex-specific differences and challenges, quantifying HAE exposure in women’s rugby league is essential to develop targeted, evidence-based strategies for injury prevention and player welfare in the women’s game.

Previous studies employing iMGs in rugby league and union typically quantify the incidence of HAEs (i.e. the rate at which HAEs are recorded for a given time denominator) during matches and the probability of HAEs during various match events (i.e. the likelihood of an HAE being recorded during events, including tackles and ball-carries). These data allow for the HAE exposure within sports to be better understood. Specifically, HAE incidence allows for HAE exposures to be estimated using exposure time [13, 25], and HAE probability allows for sporting actions associated with the greatest risk of HAEs to be identified [26–28]. Furthermore, studies in rugby union have quantified HAE incidence and probability values by position [17–19]. A more recent study in men’s rugby league quantified both position- and player-specific HAE incidence, revealing considerable variability, meaning that some players accumulated considerably greater HAE exposure per match [22]. For example, the mean HAE incidence for a centre was 1.77 HAEs exceeding 25 g per match, while one player exhibited an average incidence of 5.02 HAEs exceeding 25 g per match. If this elevated HAE incidence is replicated across a prolonged period, it could potentially represent an increased risk of neurological disorders [11, 12] and chronic traumatic encephalopathy [13, 14]. This demonstrates the importance of monitoring player-specific HAE exposure.

Therefore, the objective of this study was to examine HAE incidence and probability across WSL rugby league players, including position- and player-specific HAE incidence rates and probability.

## Methods

### Study Design and Participants

A prospective cohort study was conducted with players from all eight WSL teams during the 2024 season as part of the Rugby Football League (RFL) Tackle and Contact Kinematics, Loads and Exposure (TaCKLE) project [29]. The 2024 season was the first for iMGs to be mandated as part of the medical standards in the WSL; however, not all players wore iMGs owing to opt-out and/or medical reasons. All matches (*n* = 50) were recorded for video analysis, with two removed owing to poor quality video footage, and 136 players wore iMGs that recorded data throughout the study (Table 1). Ethics approval was received from the Leeds Beckett University Ethics Committee (ref. no. 108638).

**Table 1:** Starting positions and the number of players and player matches with iMG data

	Players (*n*)	Player matches (*n*)	Median playing time (min)
Backs			
Fullback	6	31	84
Wing	23	66	85
Centre	14	50	83
Half	11	55	84
Forwards			
Prop	18	81	55
Hooker	16	39	60
Back row	24	80	82
Loose forward	11	45	72
Interchange	13	121	42

### Instrumented Mouthguards

Prevent Biometrics (Minneapolis, MN, USA) provided all iMGs, which were custom fit, to players following digital dental scans. These iMGs were configured the same as previously described in the men’s Super League [22]. Prevent Biometrics performed various post-processing of iMG data, including the differentiation of angular velocity, kinematic filtering, transformation of linear kinematics to the head centre of gravity, and the classification of events as true positives and false positives via an in-house algorithm. Only iMG-recorded events classified as true positives by this algorithm were used in the study (*n* = 8686), as this has been shown to perform well in previous validations with positive predictive values of 0.94 [30] and 0.99 [17]. Additionally, another in-house Prevent Biometrics algorithm classified the level of noise/artefact that remained in the kinematic signal as minimal (class 0, *n* = 7619), moderate (class 1, *n* = 611), or severe (class 2, *n* = 456). Moderate or severe events were subjected to additional filters by Prevent Biometrics. This processing resulted in 50 ms periods of estimated head kinematics each time the trigger threshold was surpassed. These iMG-recorded events, sometimes termed as sensor acceleration events, were considered as a proxy measure for HAEs [15]. Therefore, while the definition of HAE, as per the Consensus Head Acceleration Measurement Practices (CHAMP) group, refers to the in vivo event [10], the term HAE is used interchangeably to describe both the iMG datum (sensor acceleration event) and the in vivo event throughout this article [15].

Following the processing by Prevent Biometrics, peak linear acceleration (PLA; g) and peak angular acceleration (PAA; rad/s^2^) were extracted from the resultant time-series data of each HAE by the research team. Only HAEs exceeding both 400 rad/s^2^ and 5 g were used to remove those triggered by non-contact events [31]. Additionally, rotational velocity change index [32] (RVCI; rad/s) was calculated owing to its high correlation with brain strain metrics [33]. The duration constraint for the RVCI equation was 10 ms, based on previous research to maximise correlation with brain strain measures [32].

### Video Analysis

Video footage with a minimum resolution of 720p and an aspect ratio of 16:9 was provided by each home team using a single camera from an elevated position on the half-way line with panning and zooming to follow the play of the ball. Any matches with video footage not meeting these requirements were removed (*n* = 2). Video analysis was conducted by two experienced rugby league analysts. This involved the coding of tackle attempts (i.e. a player attempting to halt the progression of the ball-carrier by making contact) and ball-carries (i.e. a player carrying the ball into contact with at least one tackler). This analysis provided a video timestamp and player identifier for each player involved in each tackle attempt or ball-carry event. A synchronisation process was required to align the iMG data to video footage. This was initially achieved via a cross-correlation approach [34] and later refined manually with video analysis.

### Pre-processing

To calculate HAE incidence rates, the number of individual player minutes per match was required. These data were not readily available via team sheets or video analysis; therefore, proximity sensors were leveraged for this purpose. Prevent Biometrics iMGs provide start and end timestamps of on-the-teeth periods on the basis of infrared proximity sensor data. A custom-built MATLAB function (provided in the Supplementary Material) was used to estimate playing time from these data. Briefly, the function grouped together on-the-teeth proximity periods into ‘bouts’ of iMG use, which were periods during the match when the iMG was worn and the player was an active player (i.e. on the pitch as an active player; not an interchange) (provided in the Supplementary Material). Validation of this approach included the visual inspection of estimated bouts using video analysis, resulting in the removal of 13 player matches owing to poor iMG adherence based on these visualisations. This validation was carried out for all player matches used for the calculation of incidence values (*n* = 568). Only HAEs occurring during the bout start and end times were used.

To calculate HAE probability values, the HAEs triggered by each coded tackle and ball-carry needed to be identified. Post-synchronisation event matching is an approach for identifying the event triggering each HAE from a dataset of video-coded events on the basis of their respective video timestamps. This process has been validated using men’s rugby league data; however, the different source of video analysis data used in the present study meant that separate validation was necessary. Validation was conducted on a subset of four matches from this study (*n* HAEs = 799) using the same methodology as the previous study [34]. From this subset, overall accuracy was 0.95, with a positive predictive value of 0.96 and sensitivity values of 0.99; therefore, the post-synchronisation event matching was deemed valid for use for this dataset. More detailed results of this validation are provided in the Supplementary Material.

### Statistical Analysis

#### Incidence of Head Acceleration Events

The incidence of HAEs was modelled via generalised linear mixed models (GLMMs) with a Poisson distribution and log link function. The model was fitted using the *glmmTMB* [35] package in R using player matches in which the player wore their iMG for more than 90% of their ball-carries and tackle attempts, as determined via time-synchronised proximity sensor data (*n* = 568). This was to ensure that the iMG was worn properly throughout the match. The dependent variable was the count of HAEs for each player match, while the fixed effects included player position, minutes played and their interaction. To account for the repeated measures design and potential clustering effects, random intercepts for individual players and fixtures were included. From the model, position-specific values were derived from fixed-effects coefficients of the model. Incidence values were calculated using two denominators: per full match equivalent (86 min, which was the median playing time derived from proximity sensor data for player matches in which the player played the full match; this value exceeded 80 min owing to clock stoppages throughout each match) and per median playing time (this was based on the median playing time for each position group based on the same data and reflects the typical playing time each position would play). Additionally, player-specific values were estimated for all players that recorded iMG data during at least three player matches (*n* = 71/136). These values were estimated using each player’s random intercept and their median playing time. These values reflect the typical number of HAEs each player would record per match. Separate models were fitted for a range of threshold values (*n* = 10). Thresholds were nominally selected across a range of PLA, PAA and RVCI values, consistent with previous research [22]. Only models using thresholds up to 40 g (PLA), 3000 rad/s^2^ (PAA) and 15 rad/s (RVCI) converged; therefore, these were the highest thresholds used. Confidence intervals for HAE incidence values were estimated via bootstrapping using 1000 replicates.

#### Probability of Head Acceleration Events during Tackle Attempts and Ball-Carries

The probability of HAEs occurring during tackle attempts and ball-carries was analysed via a GLMM with a binomial distribution and logit link function. The model was fitted using the *lme4* package in R and used all iMG-measured ball-carries (*n* = 6051) and tackle attempts (*n* = 12,059). The dependent variable was the binary occurrence of an HAE exceeding a given threshold (0 or 1), with the fixed effect being the type of event (tackle attempt or ball-carry). To account for individual player differences across events, a random slope for players nested within events was included. To obtain robust estimates and confidence intervals, a bootstrapping approach with 1000 replicates was conducted. Similar to the HAE incidence models, player-specific probability values were obtained via random effect for individual players with at least 20 iMG-recorded tackle-attempts and 20 iMG-recorded ball-carries (*n* = 87/136). Separate models were fitted for each threshold value (*n* = 10). Residual diagnostics of all models in the study were assessed using the performance package in R [36], and no violations of model assumptions were detected.

## Results

### Incidence of Head Acceleration Events

**Fig. 1:**
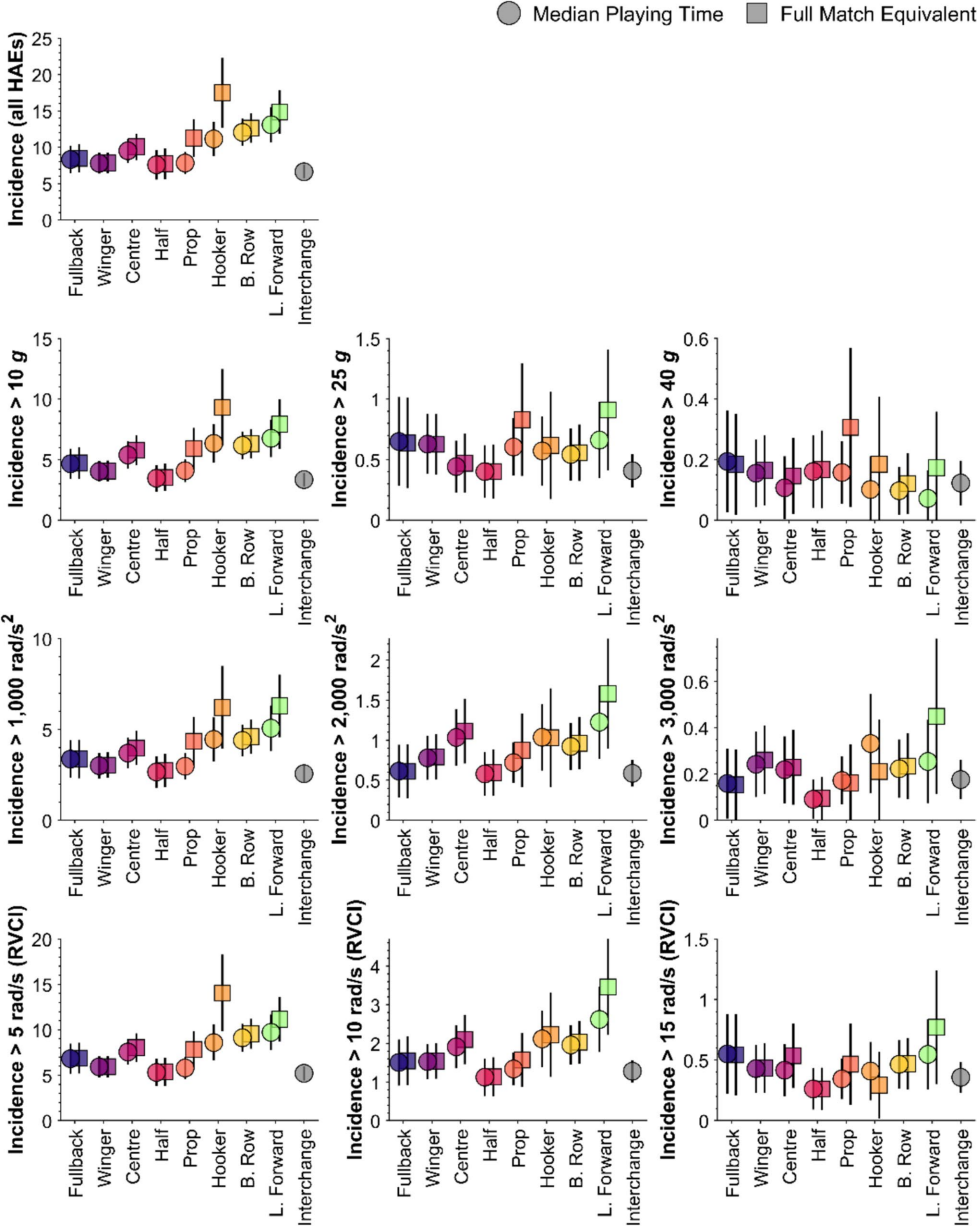
The mean incidence for each position of play using two denominators: per full match equivalent and per median playing time (i.e. the typical amount of time they played per match) using different HAE thresholds. Vertical lines behind each data point represent the 95% confidence interval (CI) for each individual player. These data are tabulated in Supplementary Table 3. *RVCI,* rotational velocity change index

Across 568 player matches, Fig. 1 shows that the incidence of HAEs per full match equivalent ranged from 7.73 to 10.06 per median playing time across back positions (i.e. fullback, winger, centre, half) and from 11.27 to 17.49 for forward positions (i.e. prop, hooker, back row, loose forward). When using a 25 g threshold, these values were lower at 0.40–0.64 for back positions and 0.56–0.91 for forward positions. When using a higher threshold, for example 40 g, HAEs were rarer, occurring in less than 12.68% (*n* = 72) of all player matches in the WSL competition. The incidence of HAEs for interchanges was 6.62 HAEs per typical match for all HAEs, 0.41 HAEs for HAEs exceeding 25 g, and 0.12 for HAEs exceeding 40 g. The peak kinematics of all HAEs are provided in the Supplementary Materials.

Across most positional groups, players typically played full matches, with the exception of hookers, loose forwards, and props, whose median playing time was 60, 72, and 55 min owing to being interchanged more frequently (Table 1). For these positional groups, incidence was higher when expressed per full match equivalent compared with when expressed per median playing time. When expressing incidence per median playing time, these values more closely reflect a typical match. For example, hookers had the highest rate of HAEs per full match equivalent (17.49); however, when accounting for how long hookers typically played for (median of 59.5 min), their exposure per median playing time was similar to other position groups (11.13).

**Fig. 2:**
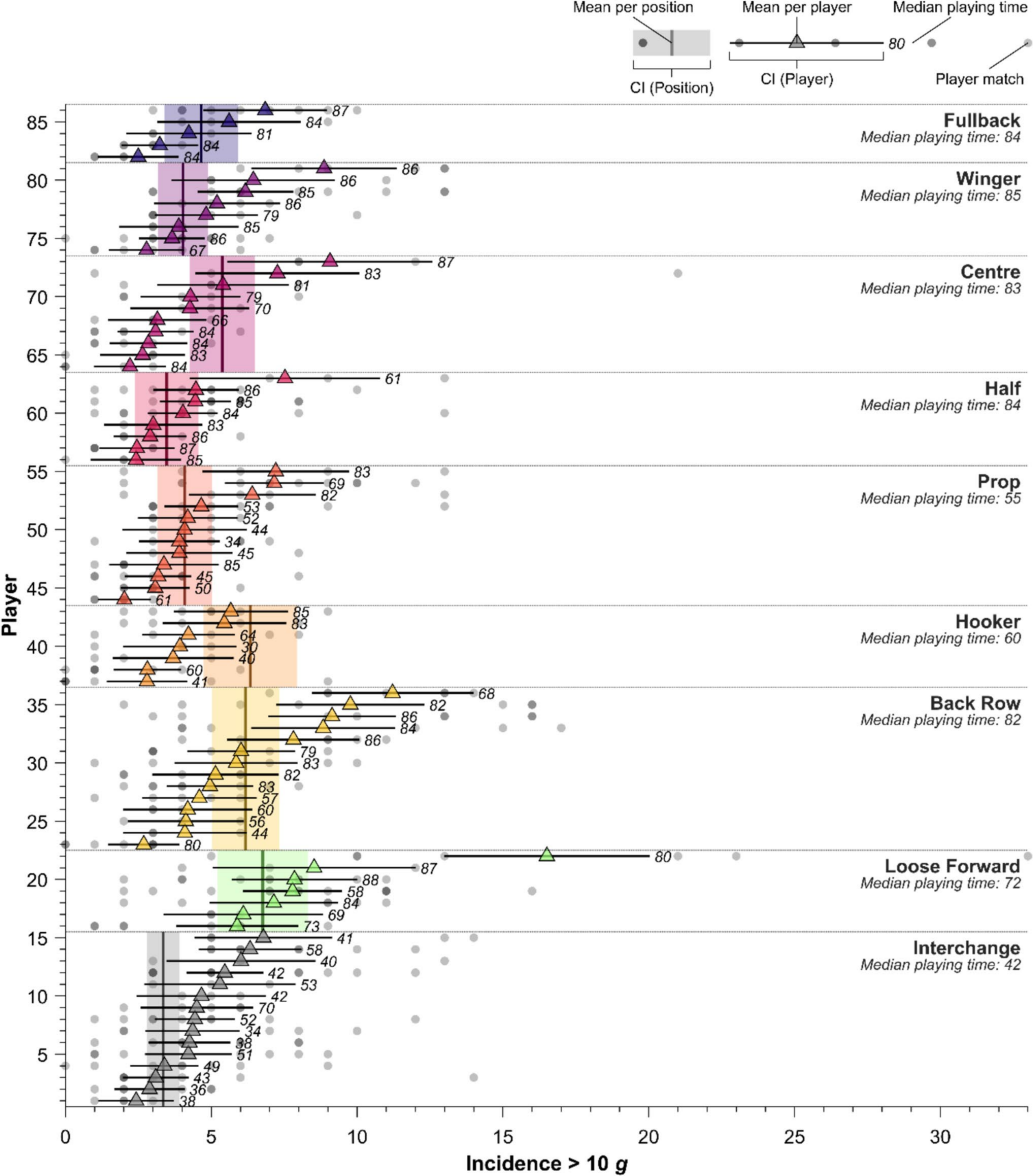
Mean incidence of HAEs exceeding 10 g per median playing time (min) for each player and each position group and individual counts of HAEs per player match. Only players with at least three player matches are included in the figure. This figure is provided with alternative thresholds in Supplementary Material 5–11. *CI*, 95% confidence interval

Figure 2 shows the incidence of HAEs exceeding 10 g per median playing time for each player with three or more iMG-recorded player matches (*n* = 71), demonstrating variability between players within each position group. For example, player 7 (loose forward) exhibited a considerably greater average number of HAEs exceeding 10 g per median playing time than other players included within the study. Furthermore, the number of HAEs this player recorded per match was also variable. Despite a mean incidence per median playing time of 16.51 (12.98–20.04) HAEs exceeding 10 g, the same player recorded 5 HAEs exceeding 10 g in one match and 33 in another.

### Probability of Head Acceleration Events

Table 2 presents the probability of HAEs exceeding a range of thresholds. The probability of tackle attempts and ball-carries resulting in HAEs was 24.39% (23.37–25.44) for ball-carries and 30.12% (28.71–31.48) for tackle attempts. When using the higher magnitude thresholds (i.e. > 40 g, 3000 rad/s^2^ or 15 rad/s [RVCI]), HAEs were rare (< 1%). Using some of the lower thresholds (all HAEs, > 10 g, > 5 rad/s [RVCI]), ball-carries had a higher probability of resulting in an HAEs than tackle attempts; however, probabilities were similar when using higher thresholds.

**Table 2:** The probability (95% CI) of an HAE exceeding a given threshold during a tackle attempt and ball-carry

Threshold	Tackle attempt	Ball-carry
All HAEs	24.39% (23.37–25.44)	30.12% (28.71–31.48)
> 10 g	13.80% (13.02–14.57)	16.99% (15.82–18.19)
> 25 g	1.28% (1.05–1.51)	1.33% (1.04–1.63)
> 40 g	0.10% (0.03–0.18)	0.13% (0.04–0.25)
> 1000 rad/s^2^	10.46% (9.76–11.13)	11.27% (10.40–12.23)
> 2000 rad/s^2^	2.28% (1.98–2.59)	1.96% (1.63–2.33)
> 3000 rad/s^2^	0.26% (0.15–0.36)	0.28% (0.14–0.42)
> 5 rad/s (RVCI)	19.58% (18.68–20.56)	23.47% (22.17–24.82)
> 10 rad/s (RVCI)	4.81% (4.36–5.27)	5.20% (4.56–5.84)
> 15 rad/s (RVCI)	0.94% (0.74–1.14)	0.84% (0.64–1.07)

**Fig. 3:**
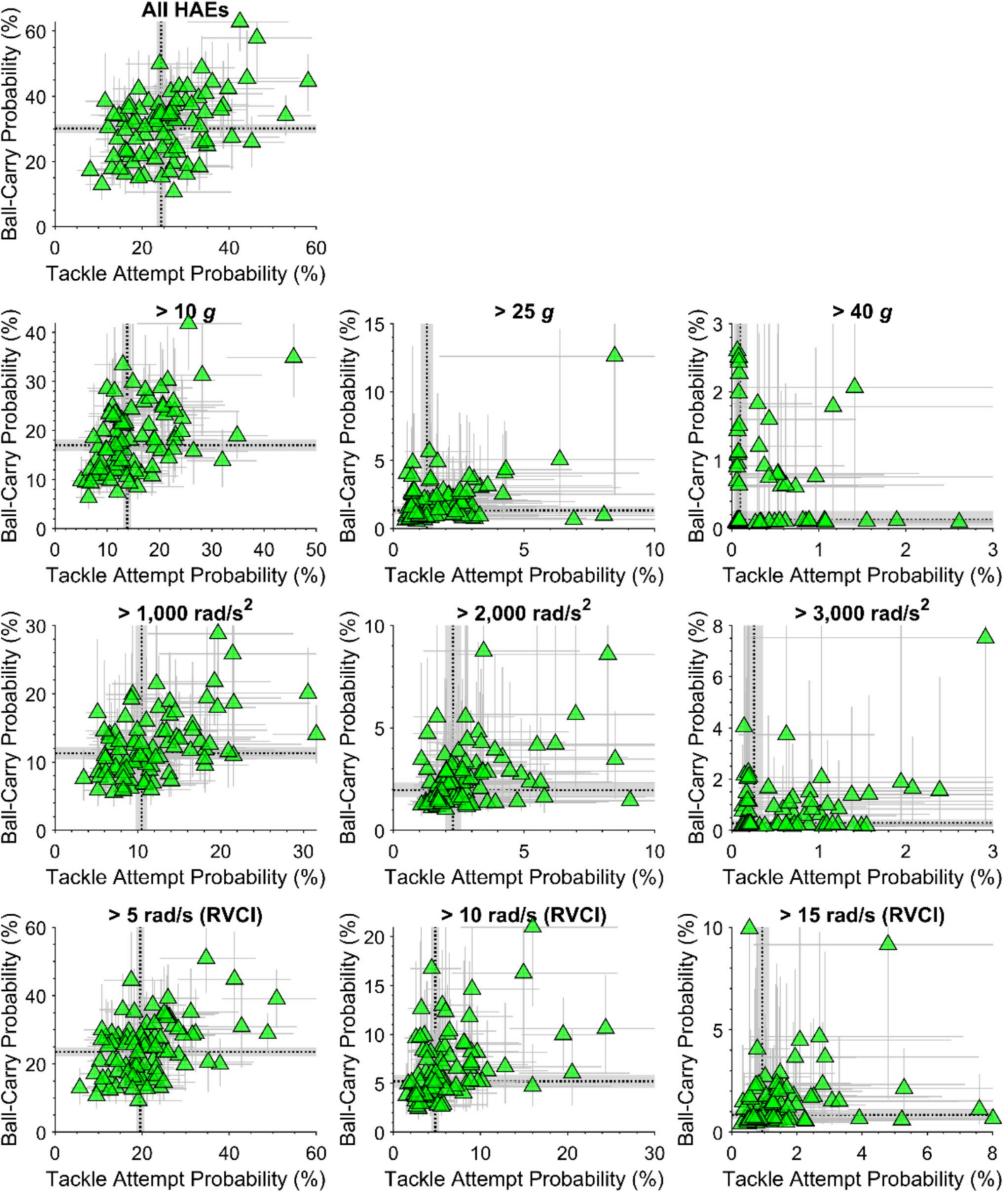
The probability of each player exceeding a range of PLA, PAA and RVCI thresholds during a tackle attempt and ball-carry. Thresholds are indicated in the individual panel titles. Players were only included if they had more than 20 tackle attempts and ball-carries (*n* = 87) to ensure sufficient data for stable and reliable estimates. Dashed lines and shaded areas indicate 95% CIs for all tackle attempts and ball-carries. Grey lines through each point indicate 95% CIs for each player. *RVCI*, rotational velocity change index

Figure 3 shows each player’s probability of recording an HAE during a tackle attempt against their probability of recording an HAE during a ball-carry. The shaded areas across the *x*-axis and *y*-axis show the average probability with 95% CIs, for ball-carries and tackle attempts, respectively. This figure demonstrates variability between players’ probabilities of recording HAEs during these match events. Some players exhibit elevated probabilities with respect to the average. For example, the average probability of recording an HAE exceeding 10 g was 13.80% (13.02–14.57) for tackle attempts and 16.99% (15.82–18.19) for ball-carries; however, several players exhibited probabilities exceeding 30%.

## Discussion

The objective of this study was to examine HAE incidence and probability across WSL rugby league players, including position- and player-specific values. The incidence of HAEs is provided using a range of magnitude thresholds. Using a 25 g threshold, HAE incidence ranged from 0.40 to 0.65 per median playing time for back positions and 0.54 to 0.66 for forward positions. The probability of tackle attempts resulting in HAEs exceeding 25 g was 1.28% and for ball-carries it was 1.33%. Finally, both HAE incidence and HAE probability were calculated on an individual basis, revealing some individuals with elevated values compared with other players. For example, one player exhibited an average of 1.77 HAEs exceeding 25 g per match, over double the average for their position. Similarly, another player exhibited a probability value over sixfold the average for tackle attempts (8.47%) and almost tenfold the average for ball-carries (12.61%) for HAEs exceeding 25 g. These findings demonstrate the importance of understanding HAE exposure on an individual basis to identify players that are at an increased risk of accumulating elevated HAE exposure and therefore warrant specific intervention.

Overall, the HAE incidence and probability values reported in the current study are considerably lower than previously reported in men’s rugby league [22] and women’s rugby union [17]. The incidence of HAEs exceeding 25 g ranged from 0.40 to 0.65 per median playing time in backs in this study, compared with 0.86–1.88 in backs in men’s professional rugby league [22]and 0.95 per full match equivalent in women’s rugby union [17]. In forwards, the incidence of HAEs exceeding 25 g ranged from 0.54 to 0.66 per median playing time in the current study, compared with 1.83–2.02 in men’s professional rugby league and 2.02 per full match equivalent in women’s rugby union. The lower HAE incidence observed in the present study may be explained, in part, by the lower probability of ball-carries and tackle attempts resulting in HAEs. The probability of exceeding 25 g in this study was 1.28% for tackle attempts and 1.33% for ball-carries, compared with 4.26% and 6.29% in men’s rugby league [22] and 4.74% and 4.55% in women’s rugby union [17]. This can also be explained by the lower number of tackle events in a typical WSL match (271 [5]) compared with a men’s Super League match (299–318 [37]). The lower number of tackle events and a lower probability of those tackle events resulting in HAEs in the WSL compared with the men’s competition may be the result of physical [38] and technical differences caused by differences in the levels of professionalism [23]. As the women’s game continues to develop, this could potentially result in increasing HAE exposure [39].

The lower average HAE incidence and probability values are encouraging. However, when quantifying player-specific HAE incidence rates and probability, some players exhibited values similar to mean values reported in men’s rugby league [22]. For example, a loose forward exhibited an average incidence of 1.77 HAEs exceeding 25 g per median playing time, threefold higher than the average for their position. Additionally, another player exhibited a probability of 12.61% and 8.47% of recording an HAE exceeding 25 g during ball-carries and tackle attempts, respectively. These probabilities are considerably higher than the mean in men’s rugby league (6.29% and 4.26% [22]) and the current study (1.33% and 1.28%). The high variation in HAE incidence and probability may be underpinned by the vast differences in anthropometric and physical qualities across WSL players, particularly between international and non-international players [38].

Elevated HAE incidence and probability values in some individuals demonstrate the importance of continually monitoring players’ HAE exposure and may support the need for individualised interventions to mitigate the potential long-term neurological risks of HAE exposure [11–14]. Understanding the mechanisms behind elevated HAE probabilities is an important future step for informing the design of such interventions. These interventions may focus on reducing the probability of HAEs during tackle events, for example, coaching interventions promoting safer tackle and ball-carry techniques, building on interventions conducted in women’s rugby league [9] and similar to interventions conducted in youth American Football [40, 41]. Alternatively, interventions may focus on reducing exposure to contact events, which may be tactical or imposed via player match limits, similar to those employed in the men’s Super League competition [42].

While these findings highlight the importance of individual monitoring and targeted interventions, it is crucial to consider how HAE exposure relates to concussion incidence in women’s rugby league. The concussion incidence in the WSL was 8–14 concussions per 1000 player match-hours [3] prior to this study; however, it decreased to 4–6 concussions per 1000 player match-hours in the 2024 season (unpublished observation), when this study’s data were collected. This rate is now lower than in the men’s Super League, which remains stable at 14.78 (95% CI 12.02–18.00) for the 2024 season (unpublished observation). The reason for this reduction is unclear; however, a lower concussion incidence relative to men’s Super League may be expected given the lower HAE incidence observed in this study [22]. Conversely, concussion rates in the WSL and women’s rugby union remain similar [8], despite higher HAE incidence in women’s rugby union [17] compared with the WSL. This discrepancy could potentially be due to differences in concussion identification and reporting, or, alternatively, may suggest that HAE risk does not always correlate directly with concussion risk.

This raises the potential that a sport, or sporting action, may simultaneously be associated with a high risk of concussion and relatively low risk of HAEs across a broader range of magnitudes and vice versa. For example, in men’s rugby league tackles, impacting the ball-carrier’s head/neck was nearly twice as likely to result in a concussion to the tackler as impacting the ball-carrier’s shoulder (odds ratio 1.88, 95% CI 1.07–3.12) [43], conversely, impacting the ball-carrier’s shoulder was more than three times as likely to result in an HAE exceeding 25 g for the tackler (probability 0.54%, 95% CI 0.28–1.00) as impacting the ball-carrier’s head/neck (probability 1.86%, 95% CI 1.54–2.23) [44]. This distinction is crucial when developing and implementing concussion and HAE-specific prevention strategies, because interventions aimed at reducing concussion rates may inadvertently increase HAE exposure as an unintended consequence. Conversely, strategies to minimise HAE exposure could potentially alter playing techniques in ways that increase concussion risk. Given these complex interactions, HAE risk and concussion risk must be monitored and detected simultaneously when implementing and evaluating interventions aimed at reducing brain injury. Furthermore, sporting actions may be associated with a low risk of concussion and indeed a relatively low risk of higher magnitude HAEs but still be associated with a high risk of lower-magnitude HAEs. These lower-magnitude HAEs may pose a separate injury mechanism themselves [45], or contribute to increased risk of concussion from subsequent actions [46, 47].

### Limitations

This is the first study to investigate HAE exposure in elite women’s rugby league using iMGs; however, there are limitations that must be considered. There are numerous limitations associated with iMG use for understanding HAE exposure and its potential risks. These have been discussed previously [15, 22]. One important limitation is the lack of understanding of the clinical effects of specific HAE magnitudes. To overcome this limitation, this study provides findings across a range of thresholds. Secondly, false negatives caused by the linear acceleration trigger bias [48] may lead to underestimated HAE incidence and probability values. This bias is unlikely to affect HAEs exceeding 25 g [15]; therefore, this threshold may be more accurate than lower-magnitude thresholds. Nonetheless, comparisons with other sports and cohorts may still be valid using other thresholds if the same iMG system and configuration is used [17–19, 42].

## Conclusions

This study quantifies the incidence of HAEs and the probability of HAEs occurring during ball-carries and tackle attempts, across a range of HAE thresholds, providing data on HAE exposure for the first time in women’s rugby league. When using a 25 g threshold, the incidence of HAEs ranged from 0.40 to 0.65 per median playing time for back positions and 0.54 to 0.66 per median playing time for forward positions. The probability of HAEs exceeding 25 g was 1.28% from tackle attempts and 1.33% from ball-carries. These values appear lower than previously reported in men’s professional rugby league [22] and women’s rugby union [17]. However, despite these relatively lower HAE incidences, some individuals exhibited elevated HAE incidence and probability values, suggesting an increased risk of accumulating excessive HAE exposure. Overall, these findings suggest that HAE exposure is lower than men’s rugby league and women’s rugby union; however, elevated HAE incidence and probability values on an individual basis support the continued use of iMGs for monitoring HAE exposure and future research to identify the mechanisms behind elevated HAE exposure in individuals.

### Policy Implications

Overall, HAE exposure appears relatively low for women’s rugby league players compared with men’s rugby league and women’s rugby union. However, elevated HAE incidence rates and probability values on an individual basis may suggest that some players are still at higher risk of accumulating considerable HAE exposure. This may necessitate the use of iMGs to identify these players for informing individualised interventions, including coaching interventions (e.g. tackle training) [40, 41] or player match-load limits [42]. Furthermore, while HAE exposure appears relatively low compared with women’s rugby union, concussion rates remain similar between the two sports [8]. Therefore, mitigation of concussion risk should continue to be a priority in women’s rugby league. During any concussion intervention, HAE and concussion risk should be monitored simultaneously to avoid altering playing techniques in ways that avoid the unintended consequence of increasing HAE risk [9].

## Supplementary Information

Below is the link to the electronic supplementary material.

Supplementary file1 (PDF 1806 KB)

## Declarations

### Patient and public involvement

Patients and/or the public were not involved in the design, conduct, reporting, or dissemination plans of this research.

### Data sharing

All data relevant to the study are included in the article or uploaded as Supplementary Information. Anonymised data should be available upon reasonable request.
