# Supplementary material for: Instrumented Mouthguards in Women’s Rugby League: Quantifying the Incidence and Probability of Head Acceleration Events at Group and Individual Levels
Source: Sports Med. 2026 Apr 10;56(8):2017–28. doi: 10.1007/s40279-026-02420-9 (PMC13457329; doi:10.1007/s40279-026-02420-9)
Supplement: Supplementary file 1 [file 40279_2026_2420_MOESM1_ESM.pdf]

Instrumented Mouthguards in Women's Rugby League: Quantifying the Incidence and Probability of Head Acceleration Events

Supplementary Material

Validation of *post-synchronisation event matching*

The *post-synchronisation event matching* method is a computational solution for identifying the triggering event of head acceleration events (HAEs) from video-coded collision events. This method as been detailed and validated in previous research[1]. Using the same methodology as this previous study, *post-synchronisation event matching* method was validated for use with the current video analysis dataset. Briefly, this validation entailed comparing outputs between manual video analysis and outputs from the *post-synchronisation event matching* method. This resulted in the classification of each HAE as a a true positive, false positive, true negative, or false negative (Supplementary Table 1). Using these classifications, diagnostic accuracy measures positive predictive value (PPV, eq.1), negative predictive value (NPV, eq.2), sensitivity (eq.3), specificity (eq.4), and accuracy (eq.5) were calculated.

Supplementary Table 1. Classification of HAEs for assessing the validity of the *post-synchronisation event matching* method.

Classification	Definition
True Positive	*Post-synchronisation event matching* and video analysis identified the triggering event of an HAE to be the same coded collision event.
False Positive	*Post-synchronisation event matching* identified the triggering event of an HAE to be a coded collision event, whereas video analysis identified the triggering event to be either a different coded collision event or an uncoded event.
True Negative	*Post-synchronisation event matching* did not identify a coded collision event as the triggering event, and video analysis identified the triggering event to be an uncoded event.
False Negative	*Post-synchronisation event matching* did not identify a coded collision event as the triggering event, but video analysis did.

$$PPV = \frac{TP}{TP + FP} \quad (1)$$

$$NPV = \frac{TN}{TN + FN} \quad (2)$$

$$Sensitivity = \frac{TP}{TP + FN} \quad (3)$$

$$Specificity = \frac{TN}{TN + FP} \quad (4)$$

$$Accuracy = \frac{TP + TN}{TP + FP + TP + FP} \quad (5)$$

A subset of four matches was used resulting in the classification of 799 HAEs. The results are provided in Supplementary Table 2.

Supplementary Table 2. The validity of *post-synchronisation event matching* compared to a manual video-analysis based approach.

True Positive	89.74% (*n* = 717)
False Positive	5.63% (*n* = 45)
True Negative	3.63% (*n* = 29)
False Negative	1.00% (*n* = 8)
PPV	0.96
NPV	0.85
Sensitivity	1.00
Specificity	0.61
Accuracy	0.95

Post-synchronisation event matching was used in the present study for the calculation of probability values (i.e., the probability of tackles and carries resulting in an HAE). *Post-synchronisation event matching* allowed for each HAE to be 'linked' to a contact event, the 'linked' HAEs were used to identify the maximum magnitude of head acceleration experienced during that contact event. For this purpose, the most important diagnostic accuracy measures are PPV (Eq.1) and sensitivity (Eq.3), because they can be used to assess the ability to correctly identify HAEs that were triggered by coded collision events; PPV evaluates the correctness of these identifications and sensitivity evaluates the ability to detect these HAEs without missing any. This validation resulted in a PPV of 0.96 and a sensitivity of 1.00, therefore the method was deemed valid for the calculation of probability.

Estimating playing time using proximity sensor data

Playing time was required as the denominator from which to calculate incidence values. However, this value was not recorded on teamsheets or during video analysis. Therefore, proximity sensor data were leveraged to estimate playing time for each player match.

Prevent Biometrics instrumented mouthguards (iMGs) are equipped with proximity sensors which are configured to provided start and end timestamps of on-the-teeth periods. These preiods indicate time periods when the instrumented mouthguard (iMG) coupled tightly with the player's teeth. Supplementary Figure 1 shows a typical proximity sensor log recorded during a player match including the timestamps of HAEs and coded tackles and carries. Of note, proximity sensor data revealed that the iMG is not placed on-the-teeth for the entirety of the match. Therefore, summing the duration of on-the-teeth would not provide an accurate estimate for playing time. However, on-the teeth periods tend to be aligned with contact events (blue circles) which indicate that players remove their iMG between contact events but wear them during contact periods.

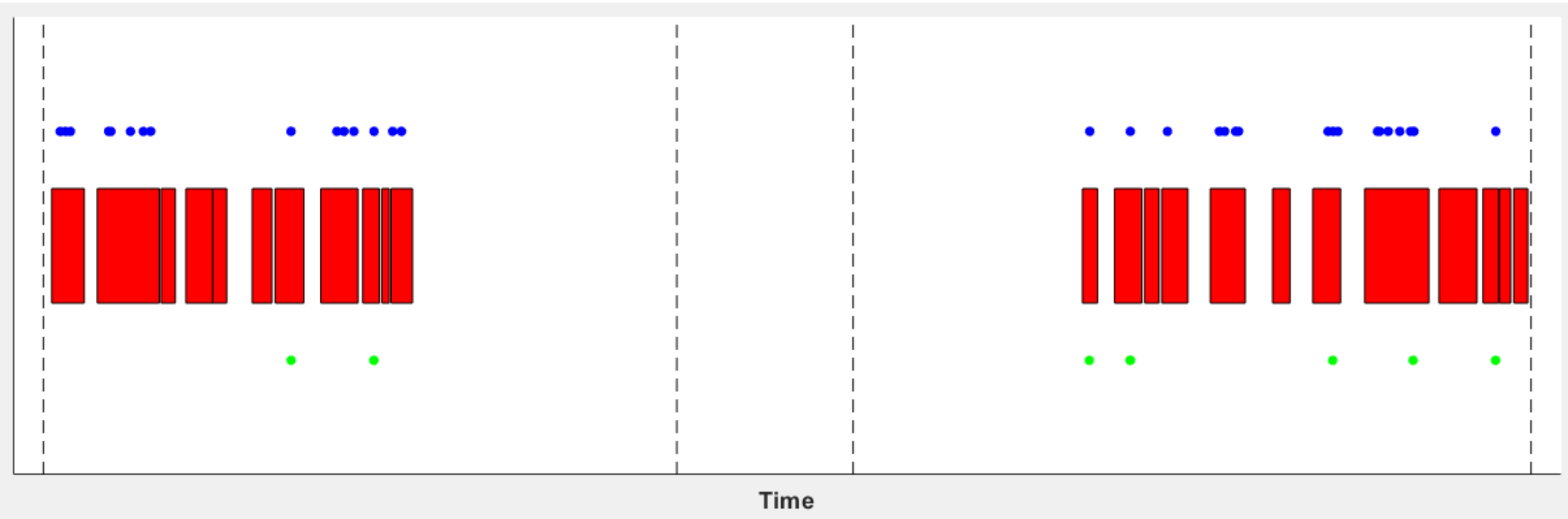

Supplementary Figure 1. On-the teeth periods (red blocks) alongside timestamps of contact events (blue circles) and HAEs (green circles). Dashed lines include the start and end periods of each half.

In order to provide an accurate estimation of playing time, various pre-processing of on-the-teeth data was required. Firstly, on-teeth-periods were synchronised to timestamps of match events (i.e., start of first half, end of first half, start of second half, and end of second half). On-the-teeth periods were then 'trimmed' to these active match periods. Secondly, on-the-teeth periods were grouped into *bouts*, which were defined as periods of play during the match where the iMG is being worn and the player is an active player on the pitch (Supplementary Figure 2). If a player played the entire game without being interchanged, they would have two *bouts*: the first and second half. The sum of the duration of all *bouts* was then used to estimate playing time for each player match. This approach was necessary because during normal play, players remove their iMGs to communicate or to take a rest, therefore a *bout* typically includes multiple on-the-teeth periods.

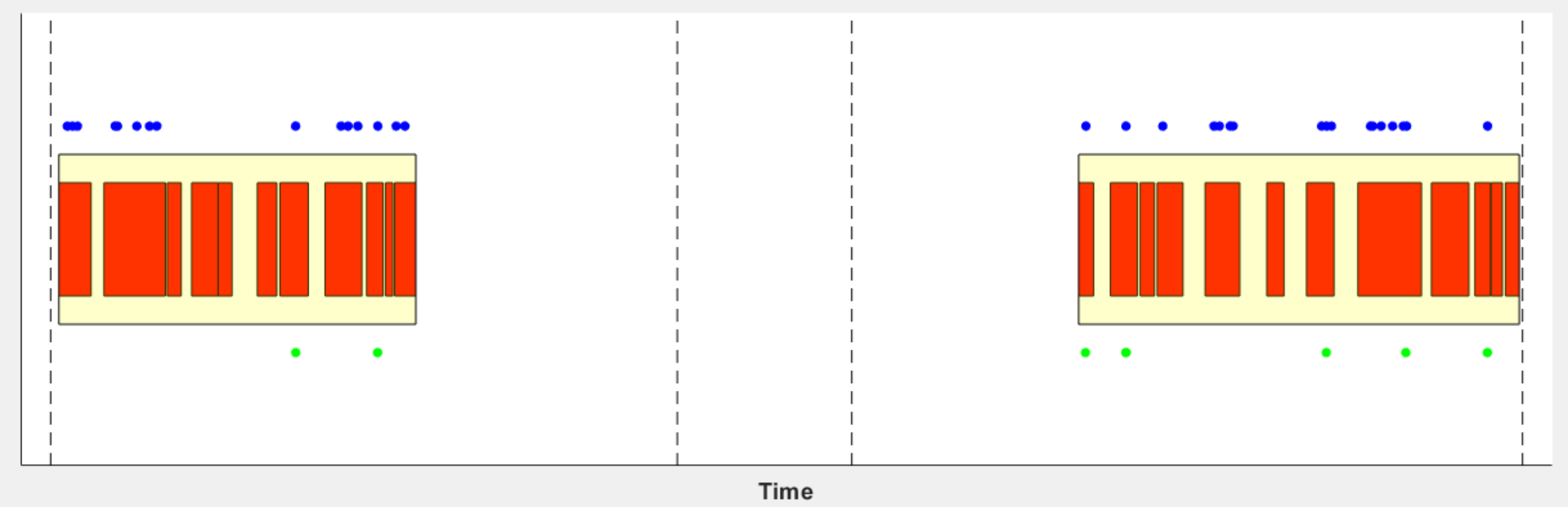

Supplementary Figure 2. Estimated time periods of *bouts* (yellow blocks) overlaid above on-the teeth periods (red blocks) alongside timestamps of contact events (blue circles) and HAEs (green circles).

The start and end point of each *bout* was estimated using a MATLAB function (Supplementary Figure 2). The function essentially groups together on-the-teeth proximity periods that meet certain criteria (like minimum duration and maximum gap between periods) to define longer *bouts* of iMG use. This function takes the start and end points of trimmed on-the-teeth periods and groups them into a potential *bout*, each potential bout is checked to see if it matches criteria that are set based on assumptions of how iMGs are worn during match play. These criteria include a maximum gap between on-the-teeth periods of less than 300 seconds (i.e., player would not typically remove their iMG for more than 5 minutes during active play), a *bout* duration of greater than 120 seconds (i.e., a *bout* duration less than this is unlikely to be active match play, for example, a player may wear the iMG for a short period while warming up or sitting on the bench), and finally a percentage of time in which the iMG is on-the-teeth. The function attemps to extend each *bout* while still meeting the aforementioned criteria, if a valid *bout* is found its start and end times are stored.

Validation of this method included the visual inspection of estimated bouts alongside the timestamps of on-the-teeth proximity periods, coded tackles and carries, and HAEs. This allowed researchers to identify that the MATLAB function (Supplementary Figure 3) correctly identified periods in which the iMG was worn and the player was involved in coded tackle and/or carry events.

```
function [bout_starts, bout_ends] = identify_prox_bouts(start_values,end_values)
    % start_values = timestamp of the start of each [trimmed] on-the-teeth period
    % start_values = timestamp of the end of each [trimmed] on-the-teeth period

    % magic numbers (criteria)
    gap_limit = 300; % no more than 5 mins without iMG on
    on_the_teeth_tolerance = 0.5; % the percentage of time the iMG must be in to be considered a bout
    minimum_duration = 120;
    minimum_initial_duration = 7.5;

    % start values
    bout_number = 1; % +1 each time we start a new bout
    start_index = 1; % the prox period at the start of the current bout
    end_index = 1;
    valid_end_index = 0;
    max_gap = 0;

    % pre-allocate outputs
    bout_starts = zeros(height(start_values),1);
    bout_ends = zeros(height(start_values),1);

    while start_index <= height(start_values) % while loop through all prox periods
        % get current bout criteria

        % initial duration
        initial_duration = end_values(start_index) - start_values(start_index);
        if initial_duration < minimum_initial_duration
            start_index = start_index + 1;
            end_index = start_index;
            continue
        end

        % gap
        if start_index == end_index
            gap = gap_limit - 1; % gap is irrelevant if one prox period
        else
            gap = start_values(end_index) - end_values(end_index-1);
        end

        if gap > max_gap
            max_gap = gap;
        end

        % bout_duration
        bout_duration = end_values(end_index) - start_values(start_index);

        % prctage_on_teeth
        bout_start_values = start_values(start_index:end_index);
        bout_end_values = end_values(start_index:end_index);
        on_teeth_duration = zeros(height(bout_start_values),1);
        for i = 1:length(on_teeth_duration)
            on_teeth_duration(i) = bout_end_values(i) - bout_start_values(i);
        end
        on_teeth_duration = sum(on_teeth_duration);
        prctage_on_teeth = on_teeth_duration/bout_duration;

        % validate current bout
        valid_bout = max_gap < gap_limit && ...
        bout_duration > minimum_duration && ...
        prctage_on_teeth > on_the_teeth_tolerance;

        if valid_bout
            valid_end_index = end_index; % store the valid_end_index
        end

        % try extending current period
        if end_index < height(start_values)
            end_index = end_index + 1;
        else % at the end
            % save the longest bout for this start_index
            if valid_end_index ~= 0 % no valid bouts
                bout_starts(bout_number) = start_values(start_index);
                bout_ends(bout_number) = end_values(valid_end_index);
                bout_number = bout_number + 1;
                % go to the next starting point
                start_index = valid_end_index + 1;
                end_index = start_index;
                % reset values
                valid_end_index = 0;
                max_gap = 0;
            else
                start_index = start_index + 1;
                end_index = start_index;
                max_gap = 0;
                valid_end_index = 0;
            end
        end
    end

    % trim output
    bout_starts = bout_starts(bout_starts ~= 0);
    bout_ends = bout_ends(bout_ends ~= 0);
end
```

Supplementary Figure 3. A MATLAB function to esimate ‘bouts’ from proximity sensor data.

The median (interquartile range) magnitude was 10.55 (8.13 – 14.63) *g* for PLA, 858.45 (616.12 – 1293.89) rad/s^2 for PAA and 6.88 (5.15 – 9.37) rad/s for RVCI (Supplementary Figure 4).

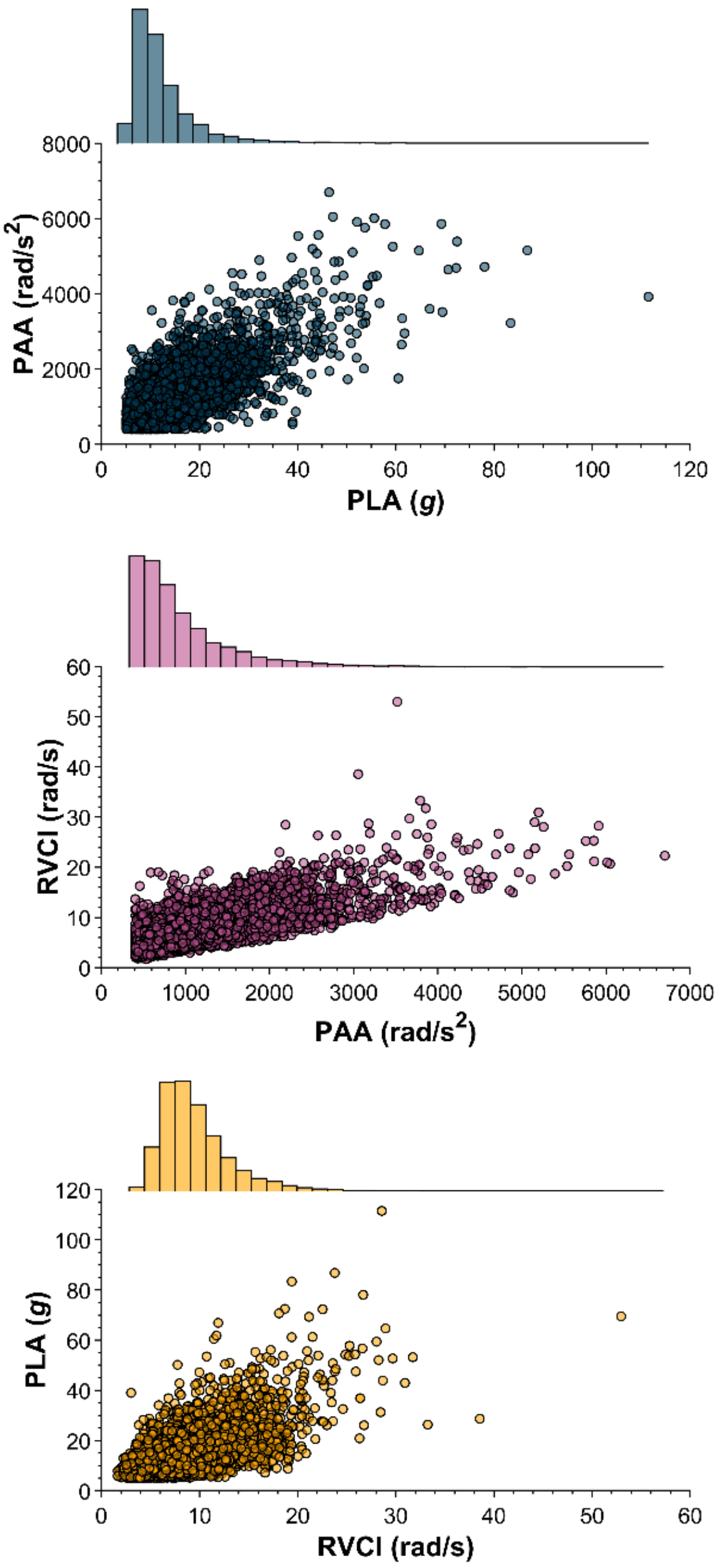

Supplementary Figure 4. The kinematics of head acceleration events classified as having minimal noise (n = 7,481). Plots are shown for relationships between PLA, PAA, and RVCI, including a distribution.

Supplementary Table 3. Tabulated incidence values (with CI) for each position group across various PLA (*g*), PAA (rad/s²), and RVCI (rad/s) thresholds. All SAEs refers to the lowest threshold that includes HAEs exceeding both 5 *g* and 400 rad/s².

Threshold	Centre	Prop	Hooker	Back Row	Loose Forward	Winger	Half	Fullback	Interchange
					Incidence per median playing time				
**All SAEs**	9.51 (7.85 - 11.16)	7.83 (6.32 - 9.33)	11.13 (8.77 - 13.50)	12.05 (10.16 - 13.93)	13.09 (10.65 - 15.53)	7.79 (6.37 - 9.22)	7.58 (5.55 - 9.62)	8.31 (6.44 - 10.19)	6.62 (5.68 – 7.56)
**> 10 *g***	5.38 (4.26 – 6.50)	4.09 (3.16 – 5.02)	6.34 (4.74 – 7.94)	6.17 (5.02 – 7.33)	6.76 (5.21 – 8.31)	4.03 (3.17 – 4.89)	3.47 (2.37 – 4.56)	4.66 (3.40 – 5.91)	3.35 (2.80 – 3.90)
**> 25 *g***	0.44 (0.23 – 0.66)	0.60 (0.37 – 0.84)	0.57 (0.29 – 0.86)	0.54 (0.33 – 0.76)	0.66 (0.35 – 0.97)	0.63 (0.38 – 0.88)	0.40 (0.18 – 0.62)	0.65 (0.28 – 1.02)	0.41 (0.27 – 0.55)
**> 40 *g***	0.11 (0.00 – 0.21)	0.16 (0.05 – 0.26)	0.10 (0.00 – 0.21)	0.10 (0.02 – 0.18)	0.07 (0.0 – 0.16)	0.16 (0.04 – 0.27)	0.16 (0.04 – 0.28)	0.19 (0.03 – 0.36)	0.12 (0.05 – 0.20)
**> 1,000 rad/s²**	3.70 (2.86 – 4.54)	2.97 (2.26 – 3.69)	4.45 (3.24 – 5.67)	4.40 (3.52 – 5.28)	5.07 (3.82 – 6.32)	3.01 (2.31 – 3.70)	2.66 (1.79 – 3.53)	3.37 (2.34 – 4.40)	2.56 (2.11 – 3.02)
**> 2,000 rad/s²**	1.03 (0.68 – 1.39)	0.72 (0.47 – 0.97)	1.04 (0.62 – 1.45)	0.92 (0.63 – 1.22)	1.23 (0.76 – 1.69)	0.78 (0.51 – 1.05)	0.58 (0.31 – 0.85)	0.61 (0.29 – 0.94)	0.59 (0.42 – 0.75)
**> 3,000 rad/s²**	0.22 (0.07 – 0.36)	0.17 (0.07 – 0.28)	0.33 (0.12 – 0.55)	0.22 (0.10 – 0.35)	0.25 (0.07 – 0.43)	0.24 (0.10 – 0.38)	0.09 (0.01 – 0.18)	0.16 (0.01 – 0.31)	0.18 (0.09 – 0.26)
**> 5 rad/s**	7.55 (6.14 – 8.97)	5.79 (4.59 – 7.00)	8.59 (6.60 – 10.57)	9.12 (7.58 – 10.65)	9.72 (7.76 – 11.68)	5.91 (4.75 – 7.07)	5.31 (3.78 – 6.83)	6.82 (5.16 – 8.47)	5.21 (4.42 – 6.00)
**> 10 rad/s**	1.91 (1.35 – 2.47)	1.34 (0.91 – 1.76)	2.11 (1.38 – 2.85)	1.96 (1.44 – 2.48)	2.62 (1.77 – 3.46)	1.53 (1.08 – 1.98)	1.12 (0.64 – 1.61)	1.50 (0.91 – 2.10)	1.28 (0.99 – 1.57)
**> 15 rad/s**	0.42 (0.20 – 0.63)	0.34 (0.18 – 0.51)	0.41 (0.17 – 0.65)	0.46 (0.26 – 0.66)	0.55 (0.26 – 0.84)	0.43 (0.23 – 0.63)	0.26 (0.09 – 0.43)	0.55 (0.22 – 0.88)	0.36 (0.23 – 0.48)
					Incidence per full match equivalent				
**All SAEs**	10.06 (8.23 – 11.88)	11.27 (8.68 – 13.85)	17.49 (12.70 – 22.28)	12.63 (10.61 – 14.65)	14.84 (11.80 – 17.88)	7.83 (6.39 – 9.27)	7.73 (5.62 – 9.84)	8.45 (6.52 – 10.39)	n/a
**> 10 *g***	5.77 (4.52 – 7.02)	5.92 (4.22 – 7.62)	9.32 (6.14 – 12.49)	6.32 (5.10 – 7.54)	7.93 (5.90 – 9.96)	4.05 (3.18 – 4.92)	3.53 (2.40 – 4.66)	4.73 (3.43 – 6.03)	n/a
**> 25 *g***	0.47 (0.23 – 0.71)	0.83 (0.37 – 1.29)	0.62 (0.17 – 1.06)	0.56 (0.32 – 0.79)	0.91 (0.41 – 1.41)	0.63 (0.38 – 0.88)	0.40 (0.18 – 0.63)	0.64 (0.27 – 1.01)	n/a
**> 40 *g***	0.15 (0.02 – 0.27)	0.31 (0.04 – 0.57)	0.19 (0.00 – 0.41)	0.12 (0.02 – 0.22)	0.17 (0.00 – 0.36)	0.16 (0.05 – 0.28)	0.17 (0.04 – 0.30)	0.18 (0.02 – 0.35)	n/a
**> 1,000 rad/s²**	3.98 (3.04 – 4.92)	4.35 (3.00 – 5.70)	6.21 (3.93 – 8.49)	4.61 (3.65 – 5.56)	6.30 (4.58 – 8.02)	3.03 (2.33 – 3.74)	2.74 (1.83 – 3.65)	3.38 (2.32 – 4.43)	n/a
**> 2,000 rad/s²**	1.11 (0.71 – 1.52)	0.87 (0.41 – 1.33)	1.03 (0.41 – 1.65)	0.96 (0.64 – 1.29)	1.58 (0.89 – 2.27)	0.79 (0.51 – 1.07)	0.60 (0.31 – 0.88)	0.61 (0.27 – 0.95)	n/a
**> 3,000 rad/s²**	0.23 (0.07 – 0.39)	0.16 (0.00 – 0.33)	0.21 (0.00 – 0.44)	0.23 (0.09 – 0.38)	0.45 (0.11 – 0.79)	0.26 (0.11 – 0.41)	0.10 (0.00 – 0.19)	0.15 (0.00 – 0.31)	n/a
**> 5 rad/s**	8.04 (6.47 – 9.60)	7.83 (5.83 – 9.83)	14.05 (9.81 – 18.29)	9.56 (7.91 – 11.21)	11.16 (8.68 – 13.64)	5.94 (4.76 – 7.11)	5.37 (3.81 – 6.94)	6.87 (5.18 – 8.57)	n/a
**> 10 rad/s**	2.10 (1.46 – 2.74)	1.57 (0.87 – 2.28)	2.22 (1.13 – 3.32)	2.03 (1.47 – 2.59)	3.46 (2.22 – 4.70)	1.55 (1.08 – 2.01)	1.14 (0.63 – 1.64)	1.55 (0.92 – 2.18)	n/a
**> 15 rad/s**	0.54 (0.27 – 0.80)	0.47 (0.13 – 0.80)	0.29 (0.02 – 0.57)	0.47 (0.26 – 0.68)	0.77 (0.30 – 1.24)	0.43 (0.23 – 0.64)	0.26 (0.09 – 0.44)	0.54 (0.21 – 0.88)	n/a

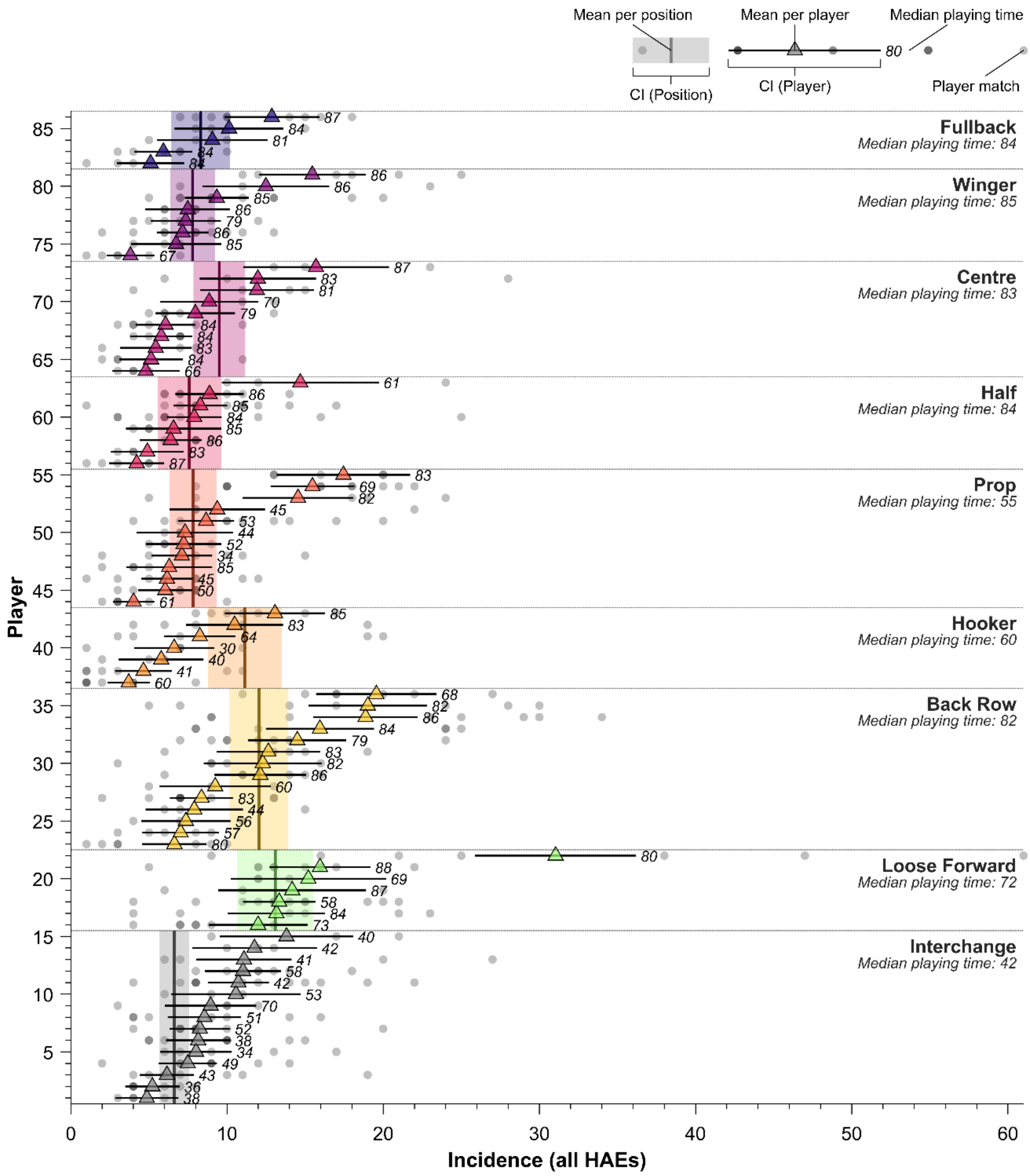

Supplementary Figure 5. Mean incidence of HAEs exceeding 5 *g* and 400 rad/s^2 *per typical match* for each player and each position group and individual counts of HAEs per player match. Only players with at least 3 player matches are included in the figure.

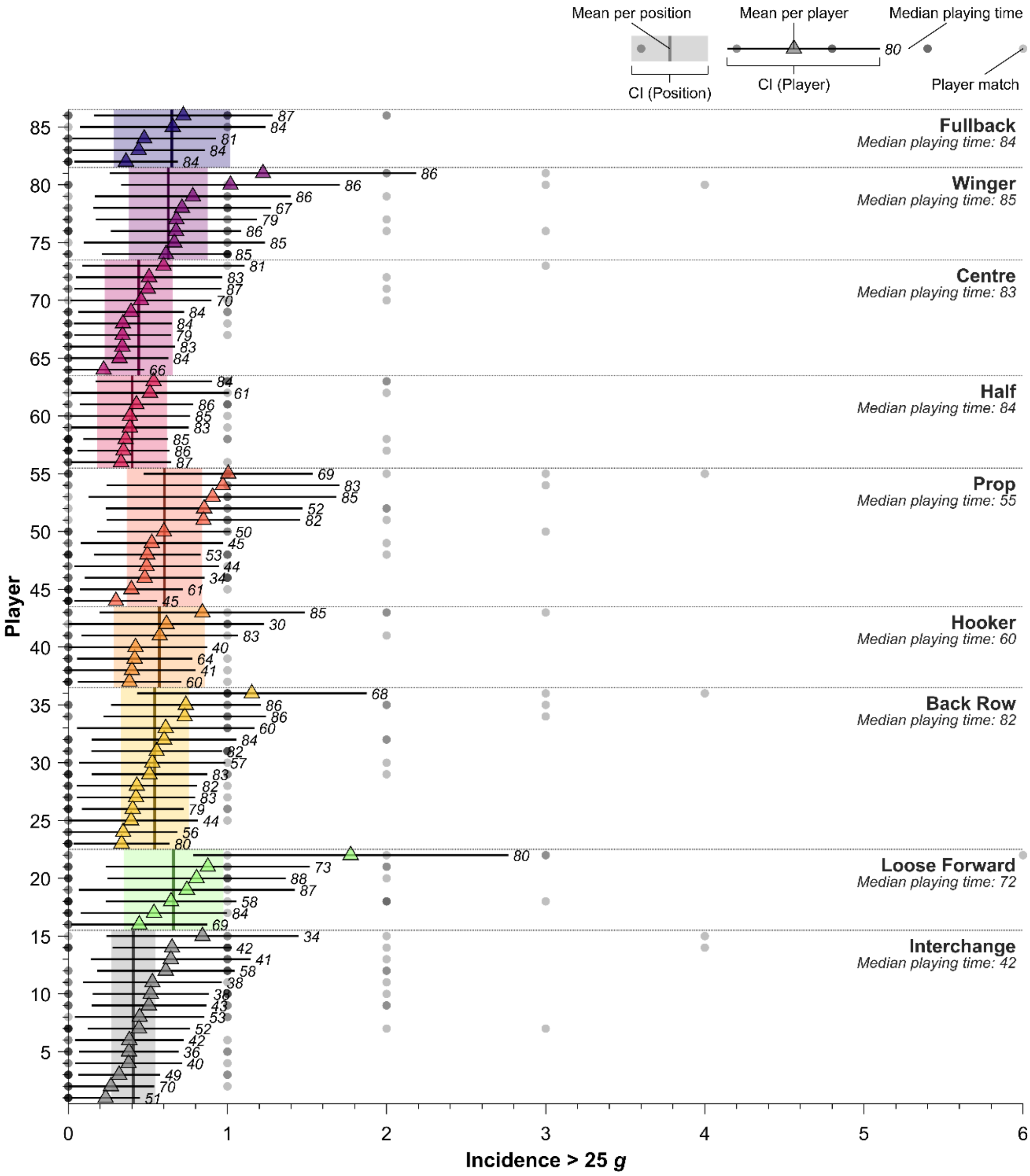

Supplementary Figure 6. Mean incidence of HAEs exceeding 25 *g per typical match* for each player and each position group and individual counts of HAEs per player match. Only players with at least 3 player matches are included in the figure.

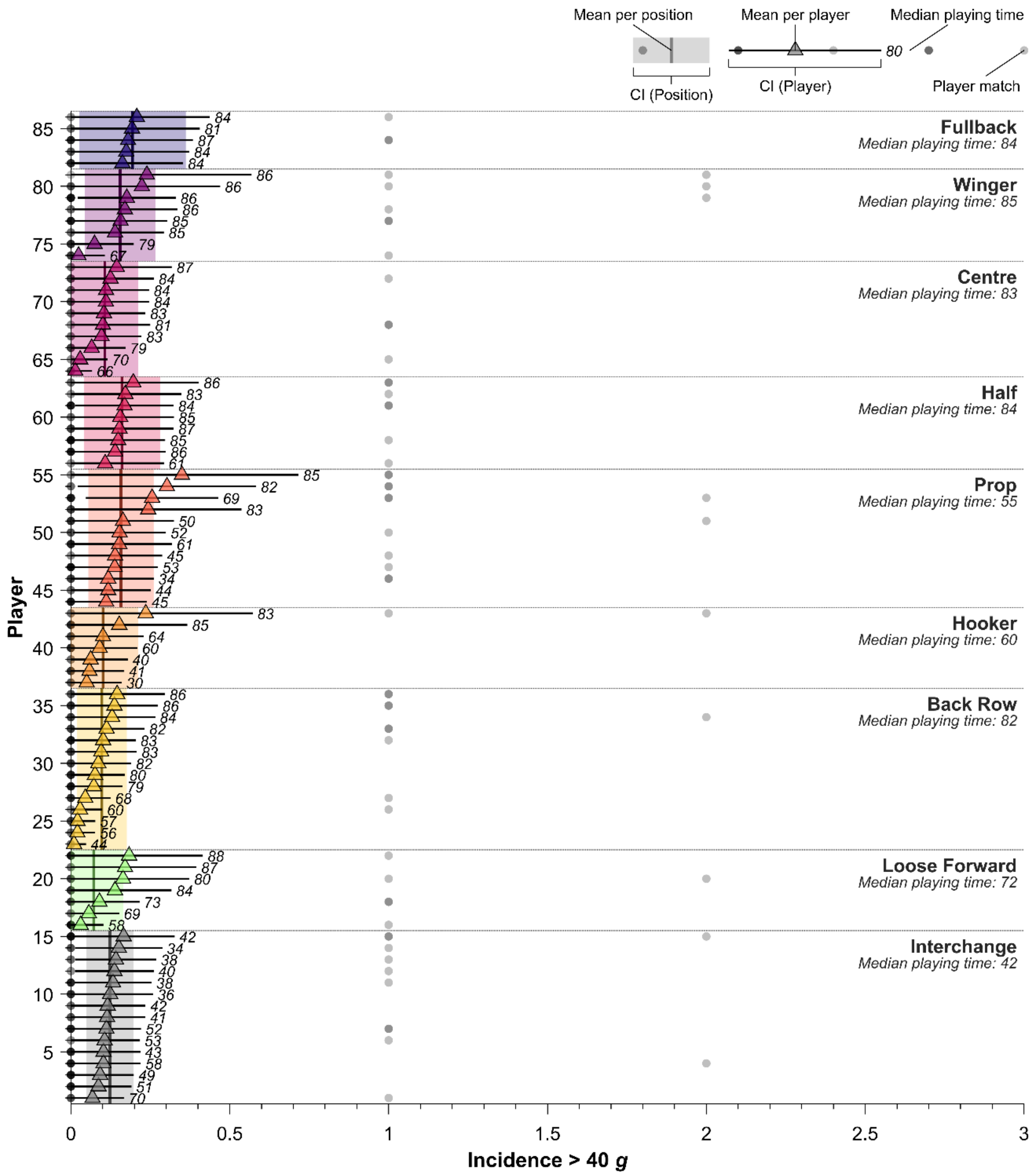

Supplementary Figure 7. Mean incidence of HAEs exceeding 40 *g per typical match* for each player and each position group and individual counts of HAEs per player match. Only players with at least 3 player matches are included in the figure.

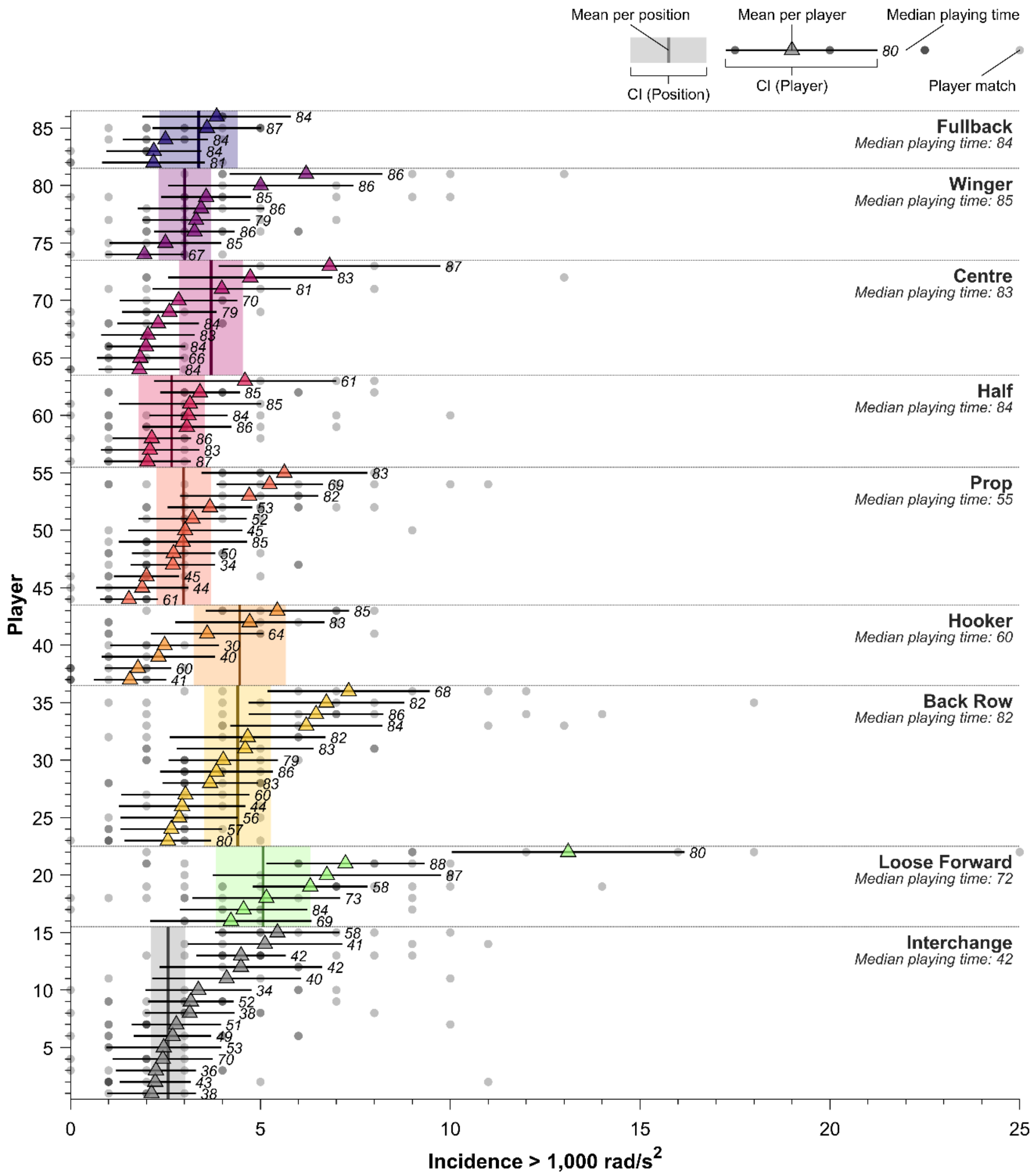

Supplementary Figure 8. Mean incidence of HAEs exceeding 1,000 rad/s^2 *per typical match* for each player and each position group and individual counts of HAEs per player match. Only players with at least 3 player matches are included in the figure.

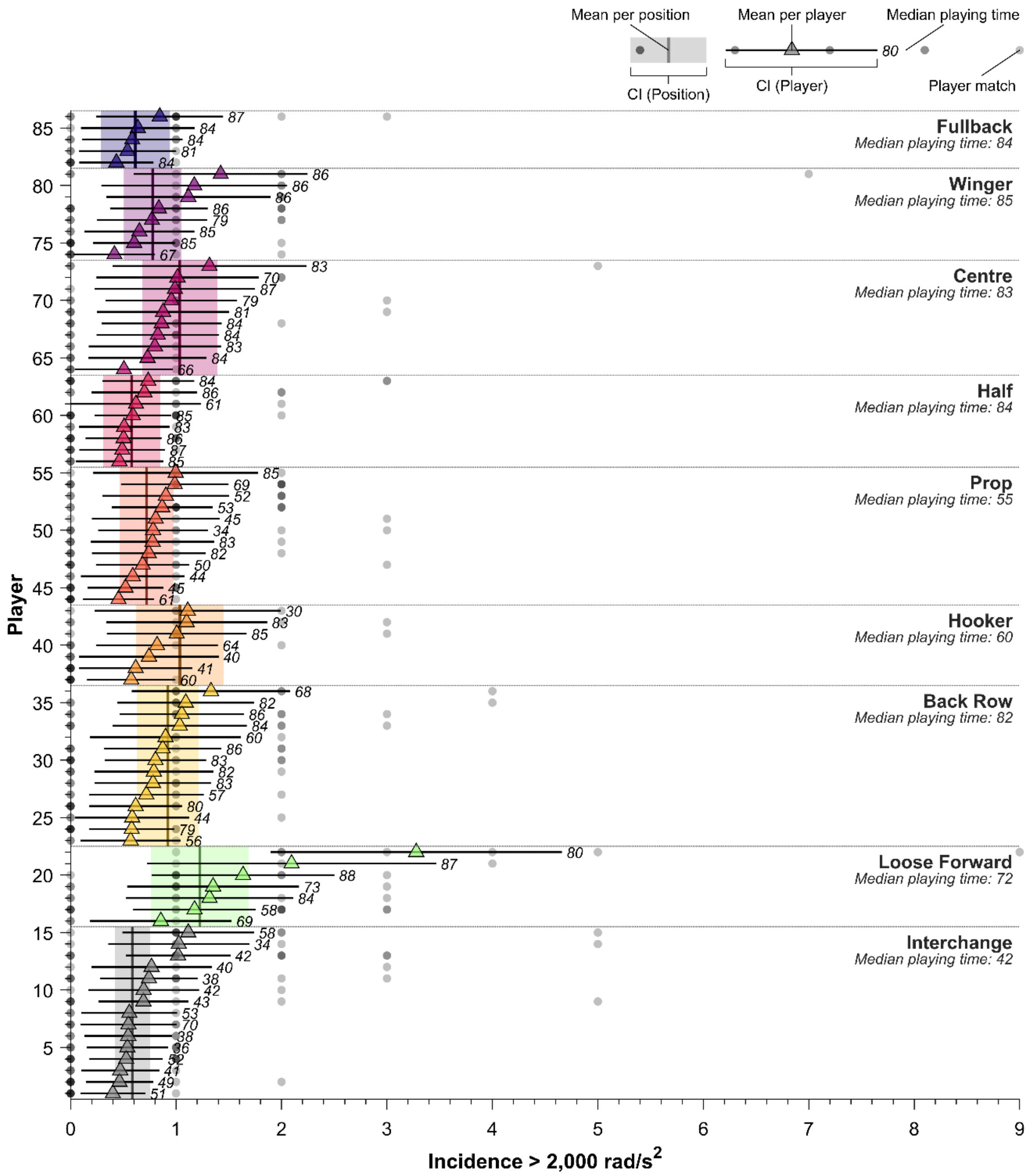

Supplementary Figure 9. Mean incidence of HAEs exceeding 2,000 rad/s^2 *per typical match* for each player and each position group and individual counts of HAEs per player match. Only players with at least 3 player matches are included in the figure.

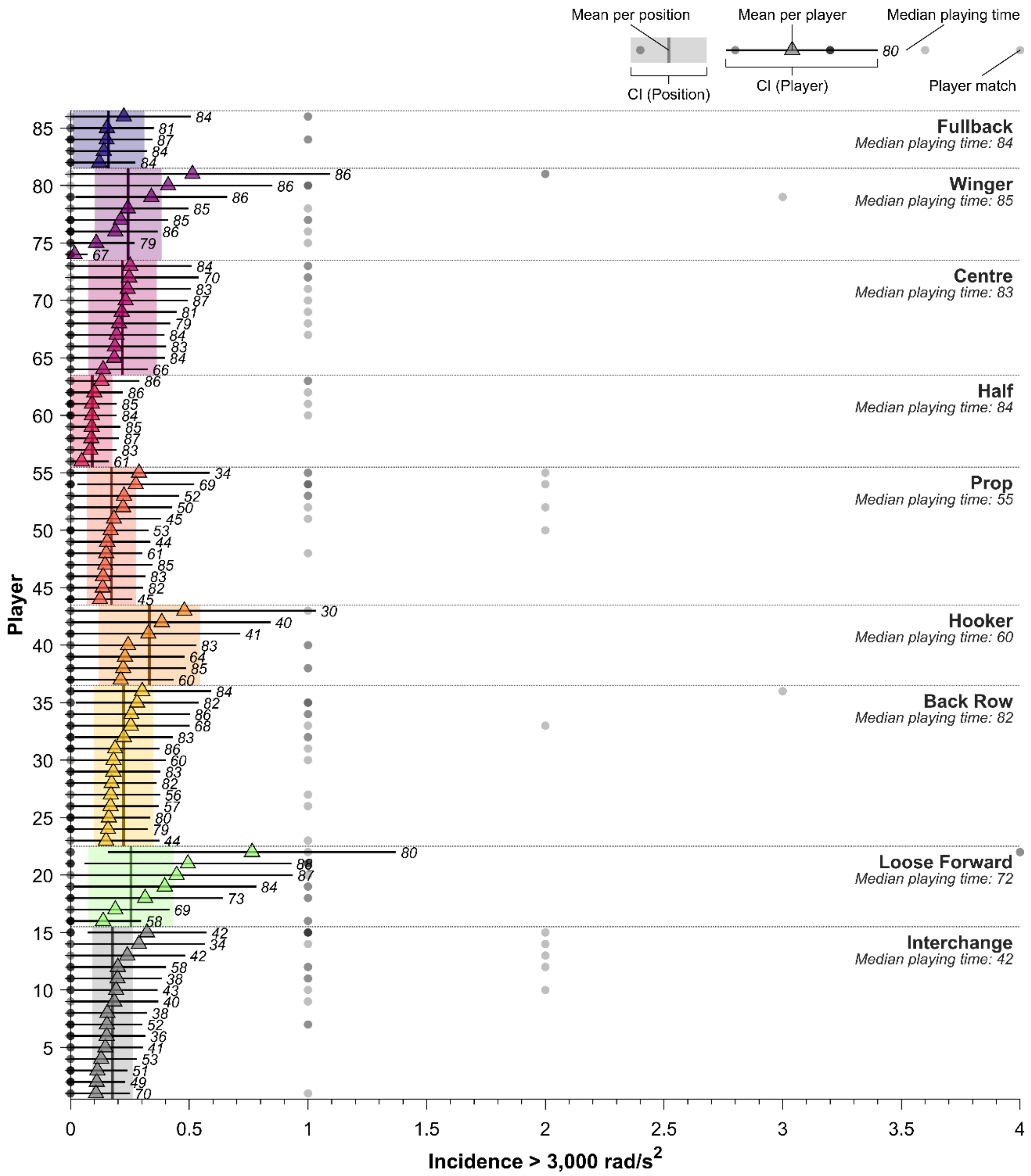

Supplementary Figure 10. Mean incidence of HAEs exceeding 3,000 rad/s^2 *per typical match* for each player and each position group and individual counts of HAEs per player match. Only players with at least 3 player matches are included in the figure.

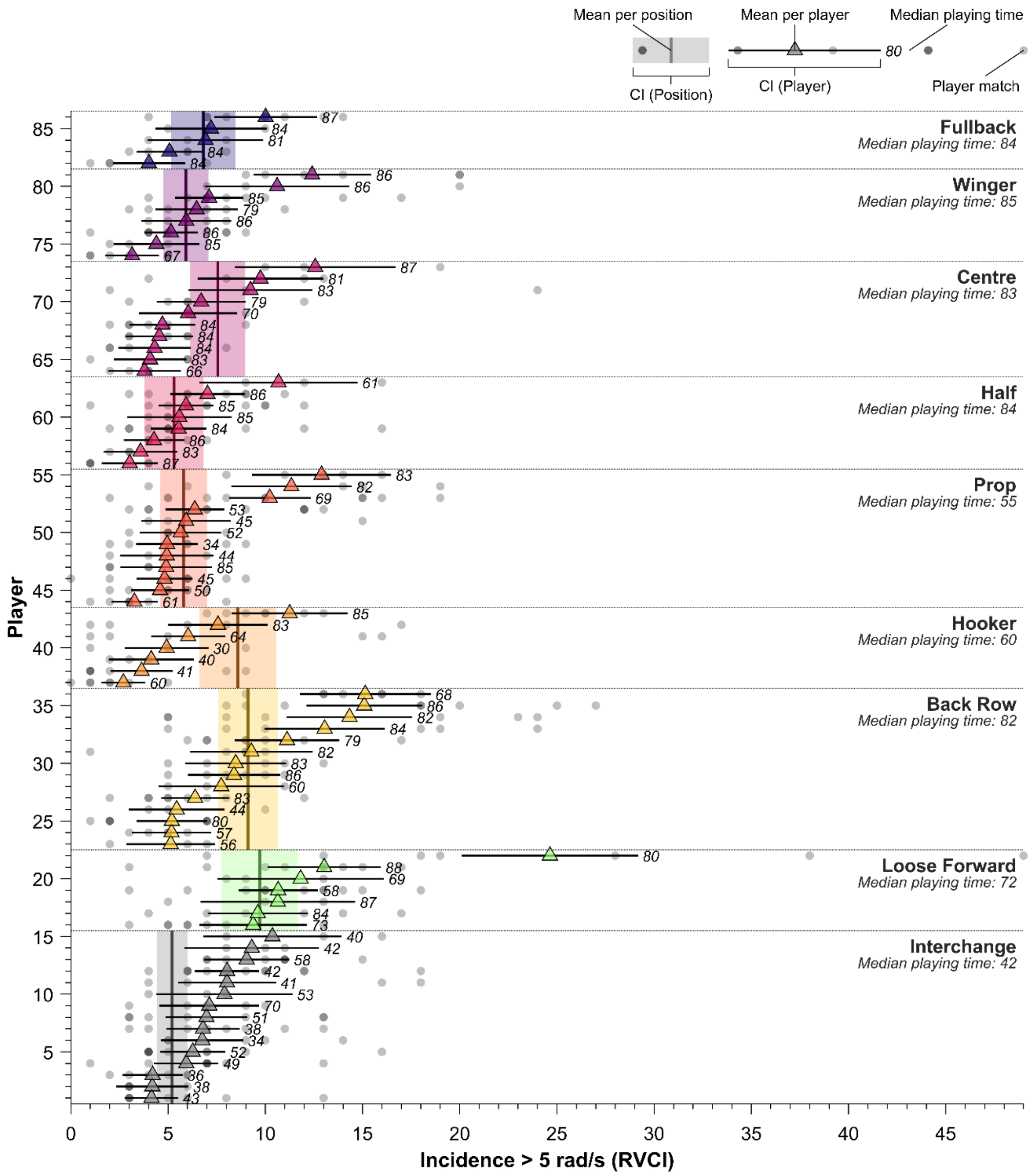

Supplementary Figure 11. Mean incidence of HAEs exceeding 5 rad/s (RVCI) *per typical match* for each player and each position group and individual counts of HAEs per player match. Only players with at least 3 player matches are included in the figure.

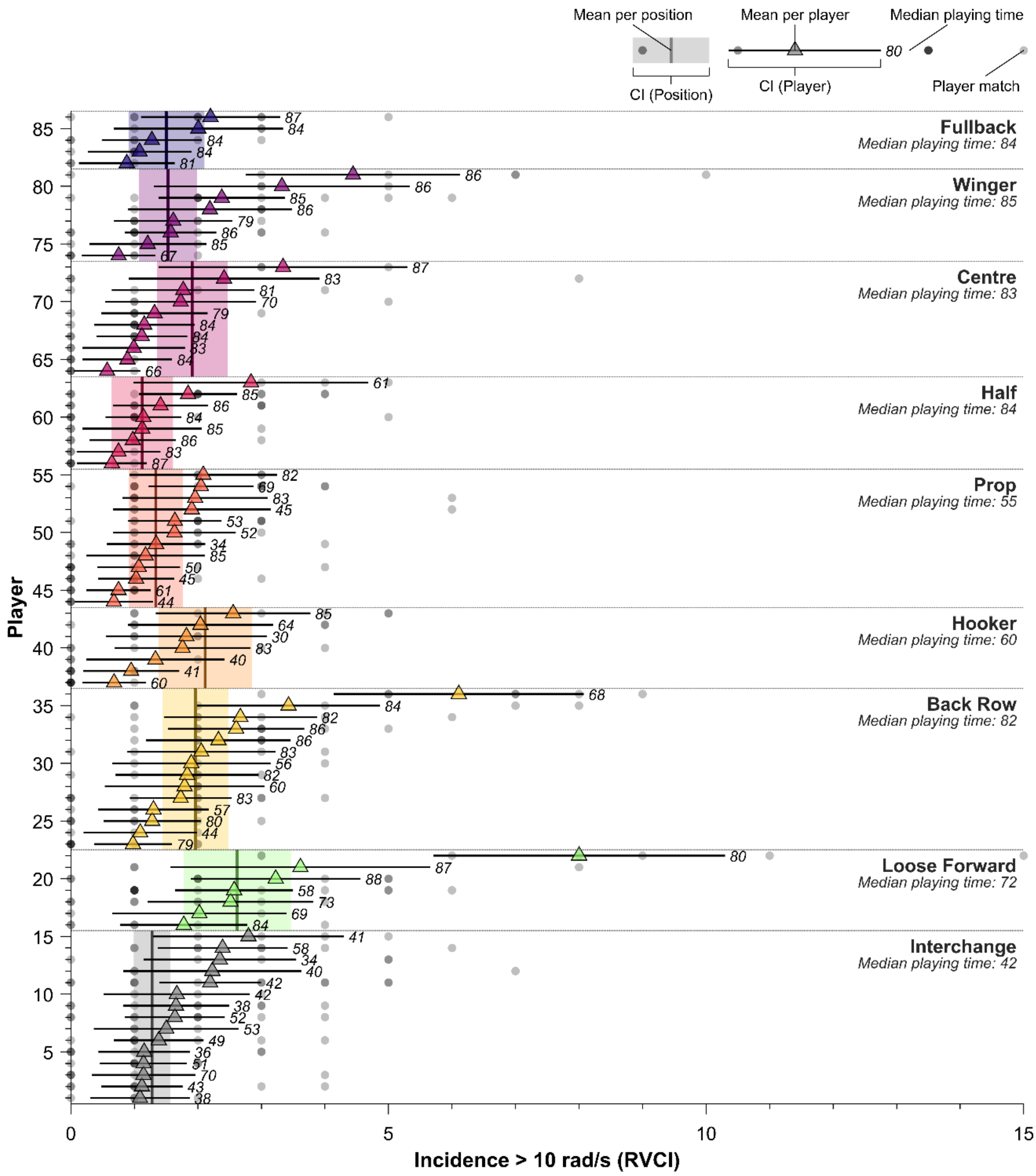

Supplementary Figure 11. Mean incidence of HAEs exceeding 10 rad/s (RVCI) *per typical match* for each player and each position group and individual counts of HAEs per player match. Only players with at least 3 player matches are included in the figure.

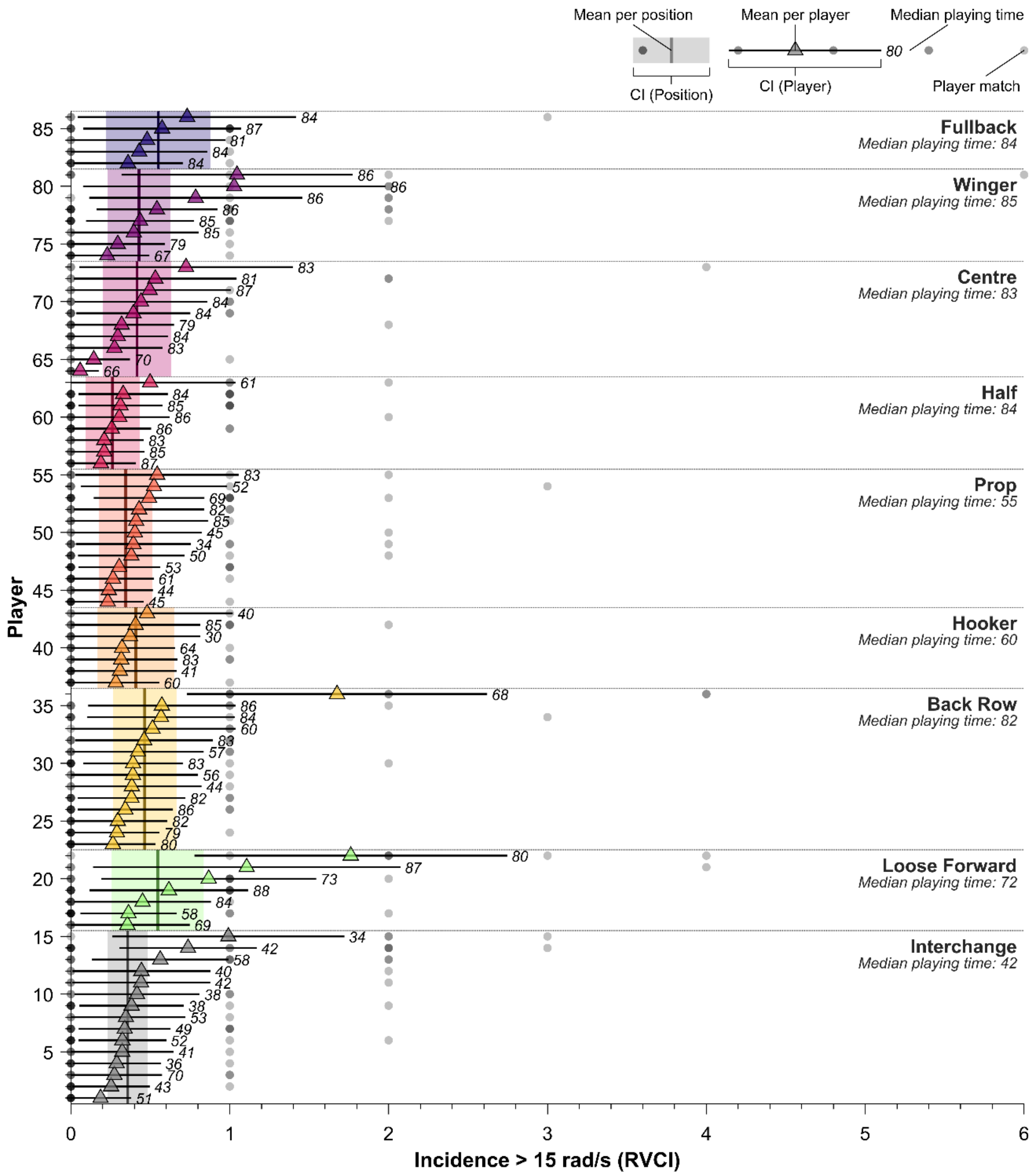

Supplementary Figure 11. Mean incidence of HAEs exceeding 15 rad/s (RVCI) *per typical match* for each player and each position group and individual counts of HAEs per player match. Only players with at least 3 player matches are included in the figure.

1. Tooby J, Rowson S, Till K, et al. Optimising Instrumented Mouthguard Data Analysis: Video Synchronisation Using a Cross–correlation Approach. *Annals of Biomedical Engineering.* 2025:1–11.
